# Detection of inflammatory bowel disease by proton magnetic resonance spectroscopy (^1^H MRS) using an animal model

**DOI:** 10.1186/1476-9255-4-24

**Published:** 2007-11-26

**Authors:** Sonal Varma, Ranjana Bird, Michael Eskin, Brion Dolenko, Jayadev Raju, Tedros Bezabeh

**Affiliations:** 1 National Research Council Institute for Biodiagnostics, Winnipeg, Canada; 2 Department of Human Nutritional Sciences, University of Manitoba, Winnipeg, Canada; 3 Department of Biology, University of Waterloo, Waterloo, Canada

## Abstract

**Background:**

The aim of this study was to analyze the potential of proton magnetic resonance spectroscopy (^1^H MRS) in diagnosing early inflammatory bowel disease (IBD).

**Methods:**

Thirty male Sprague Dawley rats were fed 2% carrageenan in their diet for either 1 or 2 weeks. ^1^H MRS was performed *ex-vivo *on colonic mucosal samples (n = 123) and the spectra were analyzed by a multivariate method of analysis. The results of the multivariate analysis were correlated with histological analysis performed using H & E stain for the presence of inflammation in the samples from each group.

**Results:**

Multivariate analysis classified the samples in their respective groups with an accuracy of 82%. Our region selection algorithm identified four regions in the spectra as being discriminatory. The metabolites assigned to these regions include creatine, phosphatidylcholine, the -C**H**_2_HC= group in fatty acyl chain, and the glycerol backbone of lipids. The differences in concentration of these metabolites in each group offer insight into the biochemical changes occurring during IBD and confer diagnostic potential to ^1^H MRS as a tool to study colonic inflammation in conjunction with biopsy.

**Conclusion:**

^1^H MRS is a sensitive tool to detect early colonic inflammation in an animal model of IBD.

## Background

Inflammatory Bowel Disease (IBD) is a common condition in North America with rising incidence in previously less affected areas worldwide [1]. It is classified as ulcerative colitis and Crohn's disease. IBD shows a bimodal peak of appearance of the disease; typically, one in the population aged 15–25 years and another peak later in life around 60 years of age [2]. Recently, it has been shown that the incidence of IBD is increasing in the pediatric population [1]. It is known that ulcerative colitis is associated with an increased risk of colorectal cancer. The incidence of colorectal cancer is estimated to be 2% in people who have had ulcerative colitis for 10 years, approaches 8% after 20 years and 18% after 30 years of having the disease [3]. As such, people diagnosed with ulcerative colitis at a younger age are exposed to a significant risk of developing colorectal cancer. IBD has a multifactorial etiology that includes an interplay of genetic, environmental and immunological factors [2]. Diet is one of the major environmental factors that is being postulated to be associated with IBD and cancer.

Dietary fat may be one of the critical determinants of pathogenesis and remission in IBD [4]. Polyunsaturated fatty acids (PUFA), specifically omega-3 fatty acids, are currently being explored for therapeutic assistance for colon cancer. Studies analyzing this effect in IBD and its transition to colon cancer are however, very limited. Recently, Meister, et al. showed that *in vitro *treatment of colon biopsies from IBD patients with omega-3 fatty acid reduced inflammation [5]. In a crossover study by Aslan et al., IBD patients on omega-3 supplements showed clinical improvement, and in 72% of them it was possible to stop or reduce the medication. However, no histopathological or biochemical improvement was observed in the patients [6]. The studies so far are controversial and have pointed towards the fact that there are many critical limitations in conducting human studies on dietary influence in IBD. Since some of these patients may be on medical therapy, having a pure placebo-control group will be very difficult. Moreover, taking frequent biopsies in these patients may not be possible.

To overcome such design limitations in studies meant to evaluate the effect of diets in the window between IBD and colon cancer, the use of an animal model that closely resembles human ulcerative colitis becomes imperative. Many chemicals have been tried to induce experimental colitis in animals [7]. Degraded lambda-carrageenan has been shown to cause inflammation similar to human IBD in a rodent model [8]. In a preliminary study (unpublished observation), our lab has shown that carrageenan can cause colitis without massive ulceration and may be suitable for long term studies on IBD as also suggested by others [9]. Based on these observations, we have used Sprague-Dawley rats fed with 2% degraded carrageenan as our model for IBD.

Another factor in conducting studies on IBD is the availability of a sensitive tool to assess inflammation. Currently, histopathological analysis is the gold standard for evaluating inflammation. As biochemical and molecular changes are known to precede histological manifestation of a disease, assessing biochemical changes would provide an opportunity to diagnose IBD at an early stage, allowing timely intervention. Magnetic resonance spectroscopy (MRS) is a technique that is extensively used to characterize the metabolic profiles of tissues both in vivo and ex vivo [10,11]. ^1^H MRS is a robust and widely used technique that identifies proton signals from various biological samples [12,13]. As proton-containing metabolites are very prevalent in tissues, ^1^H MRS is well equipped to detect the presence and relative change in concentration of these metabolites. ^1^H MR spectra show a complex array of metabolites present in the tissue, which can make the interpretation very difficult. To overcome this problem, a computer-based multivariate analysis method is being used. Multivariate analysis is particularly useful when the spectra from different experimental groups show only subtle differences in the intensity of metabolite peaks [14]. Together with ^1^H MRS, it constitutes the critical technique used in the rapidly expanding field of 'metabonomics' [15]. Bezabeh et al. have previously shown that ^1^H MRS has a potential to detect early inflammation, and also to differentiate between ulcerative colitis and Crohn's disease [10].

## Methods

### Experimental design and sample collection

Thirty male Sprague-Dawley rats (Charles River, Canada) were divided into three groups of 10 animals each. The animals in the control group were fed a regular American Institute of Nutrition-76A (AIN-76A) diet for 2 weeks; animals in the 1 week treated group were fed a regular diet for 1 week followed by 2% carrageenan (Sigma, St. Louis, MO, USA) by weight for another 1 week; animals in the 2 weeks treated group received 2% carrageenan by weight with their diet for 2 weeks. The diets were made in-house and the ingredients were purchased from Harlan Teklad, Toronto, Ontario, Canada. All the animals were sacrificed at the end of the 2^nd ^week by carbon dioxide asphyxiation. After sacrificing the animals, the colon was excised and the mucosal layer scraped off using a glass slide as described by Briere, et al. [16]. The mucosal specimens were stored in Phosphate Buffered Saline mixed with deuterium oxide (PBS/D_2_O) in cryovials and kept frozen at -80°C until processed. All the animal work was conducted at the University of Waterloo. The animal care and handling procedures were done in accordance with the Animal Ethics guidelines at the University of Waterloo and the National Research Council of Canada, Winnipeg. The mucosal specimens were coded and then shipped on dry ice to the Institute for Biodiagnostics, National Research Council of Canada, Winnipeg for ^1^H MRS and histological assessment.

### ^1^H MRS experiments

Each specimen was thawed and cut into 5–7 mm long pieces, which yielded on average 18–20 pieces. ^1^H MRS was conducted on alternate pieces, resulting in a total of 123 spectra. The samples were positioned in glass capillary tubes for ^1^H MRS experiments as described by Kuesel, et al. [17]. Briefly, the colon sample was placed in a glass capillary tube filled with PBS/D_2_O with one end plugged. This capillary was inserted into the NMR tube containing 300 μl of PBS/D_2_O solution and 5 μl of chemical shift reference, 3-Trimethylsilyl-Propionic acid-D4- sodium salt (TSP). ^1^H MRS was conducted on these samples using a Bruker Avance 360 MHz spectrometer at 25°C with presaturation of the water signal. The acquisition parameters included: 90° pulse at 9.30 μsec, number of scans = 256, spectral width = 4990.02 Hz, relaxation delay = 3 sec and time domain data points = 8 K. The samples were fixed in 10% neutral buffered formalin immediately after ^1^H MRS experiments for histological assessment.

### MRS data analysis

For multivariate analysis, each MRS spectrum was normalized by dividing the data points by the total spectral area and then aligned to the reference peak due to TSP. The region between 0.5 and 4.5 ppm was selected to eliminate the excess water signal at 4.7 ppm. First derivatives were taken, and rank ordering was done on the resulting data to eliminate baseline differences between the spectra. For control vs each of the treatment groups, random subsets of the data were used to train a genetic algorithm-based subregion selection method [18]. A histogram was constructed and the regions that stood out as being selected most often were used to develop the final classifier: linear discriminant analysis (LDA) with coefficients optimized using a bootstrapping method [19].

### Histological analysis

The formalin fixed samples were embedded in paraffin blocks and cut into 5 μm sections. The sections were stained with Hematoxylin and Eosin (H&E) stains. The slides were analyzed under a Nikon inverted microscope at 40× resolution and pictures were taken using a Nikon CoolPix 5000 digital camera. Before making paraffin blocks, the samples were coded to eliminate measurement bias. The samples were decoded only after recording the findings of histological analysis.

### Blood analysis

Blood samples were sent to the laboratory services division at the University of Guelph for complete and differential cell count. Carrageenan has been shown to induce systemic response [8], hence we speculated an increase in the white cell count in the blood of treated animals.

## Results

Multivariate analysis was performed to compare the spectra from the control group with both treatment groups. Multivariate analysis showed a significant overlap between the spectral data of the 1-week treated group and both the control and the 2-week treated groups. This indicates that after a period of 1 week, there is some inflammation in the colon, but not significant enough to set it apart from the normal colon. The analysis of the control and 2-week treated groups yielded results as shown in Table 1. All of the spectra (n = 123) were included in this analysis without correlating the data with histology.

**Table 1:** Multivariate analysis results of all MR spectra without histological confirmation (n = 123)

Control Vs. 2 weeks Carrageenan-treated group					
**Desired Class**	**N**	**Classification**		**Accuracy**	**Overall Accuracy**
				
		**Correct**	**False**		

**Control**	63	50	13	79.4%	77.2%
**Treated**	60	45	15	75.0%	

**Figure 1:**
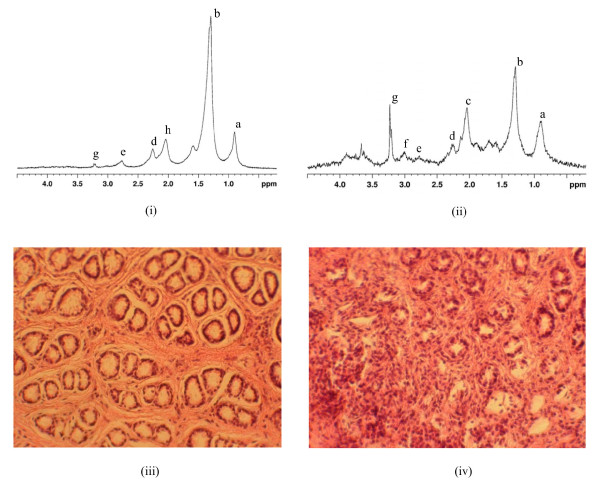
Representative ^1^H MR spectra (360 MHz) and the histology sections from the control group and 2 week-treated group; (i) MR spectrum of specimen from control group (ii) MR spectrum of specimen from 2 week-treated group (iii) H&E stained section of the same control group specimen as above (iv) H&E stained section of the same specimen from 2 week-treated group as above. The metabolites represented by the labeled peaks in the MR spectra are as follows: (a) -C**H**_3 _group of fatty acids in lipids, (b) -C**H**_2 _chain of fatty acids, (c) glutamic acid, d) -C**H**_2_-COO group of fatty acids, (e) polyunsaturated fatty acids, (f) creatine, (g) total cholines (tCho), (h)-C**H**_2_-CH= group of unsaturated fatty acids.

After conducting multivariate analysis on the complete dataset, histological assessment was performed. Figure 1 shows representative spectra from the control group and the 2 week-treated group along with the corresponding H & E stained images from the same specimens. In the histological assessment, it was observed that some samples had significant contamination by muscle tissue from the deeper layers of the colonic wall. These samples (n = 10) were eliminated from the analysis. The remaining 113 samples were evaluated for the degree of inflammation. The histological analysis of these samples revealed that some of the samples from the control group were actually inflamed and some samples from the 2-week treated group did not show any signs of inflammation. Also, it was noticed that there were differences in some of the spectral features from individual samples within the same group. This is possibly due to the fact that some of these animals did not develop any significant inflammation. In addition, the inflammation can also be patchy and may not be present uniformly throughout the colon [20]. However, we could not give a plausible explanation for colonic inflammation observed in the control group. Such spectra (n = 29) were removed from the full dataset (Table 1) and analyzed separately. Henceforth, these spectra will be referred to as 'aberrant spectra'.

After correlating with histology, the remaining 113 spectra were subjected to multivariate analysis. It was necessary to train the classifier on 'pure' spectra (n = 84) so as to distinctly identify the characteristics of inflamed and non-inflamed samples. Therefore, the aberrant spectra from both control and 2 weeks treated group (n = 29) were not used to develop the classifier for the multivariate analysis. Table 2 shows the results of the multivariate analysis of the main dataset (n = 84). The accuracy with which these samples were assigned to their respective groups increased from 77.2%, in the initial analysis, to 82%.

**Table 2:** Results of multivariate analysis with histological confirmation (n = 84)

Control Vs. 2 weeks Carrageenan-treated group					
**Desired Class**	**N**	**Classification**		**Accuracy**	**Overall Accuracy**
				
		**Correct**	**False**		

**Control**	38	31	7	81.6%	82.1%
**Treated**	46	38	8	82.6%	

The aberrant dataset (n = 29) was separately submitted to the classifier developed using the 'pure' spectra. Out of the 16 'control' samples found to be inflamed on histological assessment, the multivariate analysis classified 10 as '2 weeks treated', i.e. inflamed. Similarly, out of the 13 carrageenan-treated samples that were found to be inflammation-free on histological assessment, the multivariate analysis classified 9 as 'control'.

The reason why the four "control" samples were classified as "inflamed" by our analysis remains unclear. Further investigation needs to be undertaken on such samples to understand the exact reason for the misclassification.

One of the reasons for not attaining 100% accuracy in the MRS analysis may be the intragroup variation, particularly in the carrageenan-treated group. The degree of inflammation widely varied within the same group. Differences in intensities of metabolite peaks were observed among individual spectra from the same group of animals and in fact, from the same animal. The averaging of these variations in spectral intensities may have resulted in blunting of the effect of certain metabolites during multivariate analysis. This problem can be overcome by using a larger sample size to reduce the effect of individual variations.

Other factors that may raise concerns about the variability in the results include the use of CO_2 _asphyxiation method to euthanize the animals, leading to acute cell injury and mechanical injury during sample preparation. We felt that since we were using the same method of euthanization for all groups, this variable was common to both groups. In addition, acute hypoxic events need some time to be translated as biochemical change in the colon. The initial response to CO_2 _asphyxiation is respiratory acidosis and the brunt is on organs that are metabolically more active like brain and heart. There may also have been some tissue trauma and alteration in metabolic profile during stripping of colonic mucosa from the deeper layers. Every effort was made to minimize trauma during sample collection procedure. This procedure was performed by one person in an effort to keep the processing time and force reasonably uniform for all the samples.

Our region selection algorithm identified 4 regions of the spectra that were most discriminatory, i.e. were selected most often by the genetic algorithm. These included spectral resonances due to the -C**H**_2_HC= group in fatty acyl chain of triglycerides (2.79–2.83 ppm), creatine (3.00 ppm), phosphocholine (3.56–3.60 ppm) and glycerol backbone of lipids (4.03–4.05 ppm).

## Discussion and conclusion

Martin et al. have characterized the metabolic profile of rodent intestinal tissue including proximal and distal colon using high resolution ^1^H MRS [21]. They observed significant levels of phosphatidylcholine, creatine, glycerol and triglycerides in the normal colon. Phosphatidylcholine is the predominant phospholipid present in the cell membranes [22] including colonic mucosal cells [23]. It is also an important constituent of the inflammatory mediator, platelet activating factor (PAF). PAF is crucial for critical steps in inflammatory cascade such as platelet activation and increase in vascular permeability. The active species of PAF have been shown to increase significantly in mucosal biopsies from ulcerative colitis patients [24]. PAF levels in stool are considered diagnostic and indicative of severity in patients with ulcerative colitis [25]. The increased levels of phosphatidylcholine seen in our study may be because of increased levels of PAF in colitis. Increased apoptosis and cell turnover in IBD may also contribute to increased phosphatidylcholine concentration in colonic mucosa.

Creatine is a metabolite present in normal tissues and serves as an energy reservoir. It is converted via a reversible reaction into phosphocreatine by adding a high energy phosphate bond in the presence of creatine kinase. Phosphocreatine is broken down to creatine while releasing energy as ATP whenever required. Significant concentrations of creatine have previously been detected in human colonic mucosa [26]. The BB isoenzyme of creatine kinase has been isolated from normal [27] and infracted colonic mucosa [28]. As colonic inflammation causes cellular damage and increased requirement for energy, the creatine concentrations may be elevated in these cells.

The region around 2.8 ppm denotes the peak from protons in polyunsaturated fatty acids (PUFA) [29]. PUFAs are critical for maintaining the membrane structure and function of various cells in the body [30]. Linoleic acid is an important constituent of the lipid bilayer of cell membranes and a precursor for another PUFA, arachidonic acid. Arachidonic acid, in turn, is the precursor of potent inflammatory mediators such as LT-B4 and PG-G2.

These inflammatory markers are increased in many disorders in the human body including IBD [31]. Nishida et al., observed an increase in arachidonic acid concentration in the mucosa of patients with ulcerative colitis [32]. Nieto et al. also observed an increase in linoleic acid concentration in the colonic mucosa of rats 2 weeks after inducing experimental colitis [33]. Increase in glycerol resonance in our samples may be reflective of an increase in the total lipids in the colonic mucosa during inflammation. These changes in colonic mucosal lipid profile are similar in human ulcerative colitis as well as experimental colitis in animals.

It should be noted that the above metabolites are seen in normal as well as diseased states in colonic mucosa. The difference in their intensities is what is more significant and indicative of a disease process as opposed to only the presence or absence of these metabolites. The change in the overall profile of the spectrum, and not changes in the intensity of only one or two specific peaks, is a better indicator of the disease process.

To assess the systemic effects of colonic inflammation, we analyzed blood samples from animals in the control (n = 5) and 2 week-treated group (n = 6). As IBD is a chronic inflammation, we compared the lymphocyte counts in these animals. In 10 out of these 11 animals, the histological assessment of inflammation agreed with their respective lymphocyte counts. In one animal, the samples were classified as inflamed by histology whereas the lymphocyte counts were not raised. Since the samples from this animal were kept in the refrigerator for longer period of time than any other sample (over 2 days), some changes might have occurred in the tissue specimens interfering with the results of the histological analysis.

The results of this study need to be further reinforced by correlating the findings of ^1^H MRS with systemic markers of inflammation such as leukotriene B4 levels, serum C-reactive protein, albumin and thrombocytosis. In addition, testing the reproducibility of these results in other models of colonic inflammation such as IL-10 knock-out mice and dextran sulphate-induced colitis model will help validate this new role of ^1^H MRS. Moreover, there may be critical differences in the degree and chronicity of inflammation in different parts of the colon. Based on the results of this study, further work needs to be undertaken to examine the differences in inflammatory profile between distal, middle and proximal colon.

In conclusion, this study demonstrates that ^1^H MRS combined with multivariate analysis is a highly sensitive technique to distinguish between inflamed and non-inflamed colonic tissue. In addition, it also suggests that ^1^H MRS can serve as a tool to study the sequential progression of IBD and explore the effect of various dietary and therapeutic agents on this process. This model will be helpful to approach a multitude of questions like whether and how dietary practices can affect the development and progression of IBD. It will also enable us to overcome some of the limitations faced in human studies.

## Competing interests

The author(s) declare that they have no competing interests.

## Authors' contributions

**SV **conducted ^1^H MRS experiments and histological procedures including reading of the slides to assess inflammation. She also did pre-processing of MR spectra and drafted this manuscript. **TB **contributed to the conception of the study, designing of MR experiments and analyzing the results. He was involved with critical evaluation and revisions of the manuscript to give it the final shape. **RB **was also involved with the conception of the study, entire animal work and sample collection. Her revisions of the manuscript and suggestions were crucial for drafting this manuscript. **ME **was involved with the conception of the study, interpretation of data and revision of the manuscript. **BD **performed multivariate analysis of MR spectra and revision of the manuscript. **JR **was involved with animal work and colon sample collection. All authors have read and approved the final version of this manuscript.
